# Effects of focal low energy extracorporeal shock wave treatment on reduction of sialorrhea in Parkinson’s disease

**DOI:** 10.3389/fneur.2025.1740286

**Published:** 2026-01-06

**Authors:** Paolo Manganotti, Sophie Rangan, Mauro Catalan, Arianna Sartori

**Affiliations:** 1 Neurology Unit, Hospital Care Department of Medicine, Azienda Sanitaria Universitaria Giuliano Isontina, Trieste, Italy; 2 Neurology Unit, Department of Medical, Surgical and Health Sciences, University of Trieste, Trieste, Italy

**Keywords:** ESWT, Parkinson’s disease, shock wave therapy, sialorrhea, toxin

## Abstract

**Introduction:**

Sialorrhea is a frequent and disabling non-motor symptom in Parkinson’s disease (PD) and atypical parkinsonian syndromes, often exerting a serious impact on patients’ health and quality of life (QoL). Botulinum toxin injections into the major salivary glands, which reduce salivary secretion, represent an established and effective therapy. However, even with adequate operator training and ultrasound guidance, transient dysphagia may occur. Low-energy extracorporeal shock wave therapy (ESWT) is a noninvasive neuromodulatory technique that has shown anticholinergic-like and antispastic effects in neurological disorders.

**Aims:**

To evaluate the effects of low-energy ESWT applied to the salivary glands as a potential treatment for sialorrhea in patients with PD or atypical parkinsonian syndromes.

**Methods:**

In this pilot observational study, 12 patients with advanced PD or atypical parkinsonism and clinically significant sialorrhea (UPDRS-II item 2 ≥ 2) underwent two weekly sessions of bilateral focal ESWT (750 impulses per gland, 0.1–0.3 mJ/mm^2^, 5 Hz) under ultrasound guidance. Clinical assessments were performed at baseline and at 1st, 4th, 8th, and 12th week post-treatment using the Drooling Frequency and Severity Scale (DSFS, primary endpoint), Nocturnal Hypersalivation Rating Scale (NHRS), Sialorrhea Clinical Scale for PD (SCS-PD), Drooling Impact Score (DIS), and both Clinical and Patient Global Impression of Change (CGI-C, PGI-C).

**Results:**

A statistically significant improvement was observed across all scales at week 4 post-treatment, with more sustained effects on DSFS and SCS-PD. Improvements were evident as early as week 1 and persisted up to week 8 for SCS-PD. DIS scores significantly decreased from baseline to week 4 (*p* = 0.005). Subjective improvement was reported by 75% of patients (PGI-C) and confirmed by clinicians in 83.3% (CGI-C). No adverse effects, pain, or dysphagia were reported.

**Conclusion:**

Focal low-energy ESWT demonstrated efficacy in reducing sialorrhea in patients with advanced PD and atypical parkinsonism. These preliminary findings suggest a novel, well-tolerated, and noninvasive treatment approach that may improve QoL and serve as a potential alternative or adjunct to botulinum toxin therapy.

## Introduction

Sialorrhea is defined as the chronic and excessive flow of saliva beyond the lip margin, which may substantially impair both social functioning and overall quality of life (QoL) (1, 2). Excessive salivation represents a frequent yet often underestimated complication of several neurological disorders, most notably cerebral palsy (CP), Parkinson’s disease (PD), and amyotrophic lateral sclerosis (ALS) (3–6). Reported prevalence in PD varies across studies but frequently exceeds 50%, reflecting differences in disease stage, assessment methods, and populations (7). Rather than resulting from excessive salivary gland secretion (hypersalivation), sialorrhea in PD usually results from impaired oropharyngeal motor control or swallowing dysfunction, leading to inefficient saliva clearance and accumulation in the oral cavity (8–10). Current treatment options include pharmacological agents, often used off-label, as well as conservative non-pharmacological interventions. Systemic drugs, particularly anticholinergic agents, are frequently associated with adverse effects (11, 12). In contrast, intraglandular botulinum neurotoxin type A injections (IncoBoNT/A) exert their effect locally at the site of saliva production, offering a focal and well-tolerated alternative. IncoBoNT/A has been approved for both adult and pediatric patients, representing a valid option or adjunctive therapy (13). Recent clinical trials and subsequent regulatory approvals have confirmed the efficacy of IncoBoNT/A for sialorrhea treatment, validating long-standing clinical observations (14–24). However, botulinum toxin injections are associated with certain limitations and risks, largely due to their invasive nature. The procedure requires specific preparation, operator training, and clinical experience. Ultrasound guidance is generally recommended to improve accuracy and safety (25, 26). However, excessive or improperly targeted injections in the submandibular gland may result in transient but clinically significant dysphagia, particularly in patients with advanced PD (13, 14, 20, 21, 27). Conversely, insufficient dosing may fail to adequately control symptoms. Moreover, the invasive nature of the procedure poses challenges in patients with CP, where tolerance and acceptance are often limited. Given these limitations, alternative noninvasive approaches warrant investigation.

Low-energy extracorporeal shock wave therapy (ESWT) has recently emerged as a noninvasive neurological intervention primarily employed to reduce spasticity and hypertonia in conditions such as stroke and CP (28–30). Preclinical and clinical studies suggest that ESWT may transiently reduce muscle hypertonia through mechanisms involving decreased acetylcholine release at the neuromuscular junction, enhanced nitric oxide (NO) release, and anti-inflammatory effects (31, 32).

The aim of this study was to investigate the effects of low-energy, noninvasive ESWT applied to the salivary glands in patients with advanced PD or atypical parkinsonian syndromes and persistent sialorrhea, with the objective of assessing its potential anticholinergic-like action and evaluating its efficacy in reducing hypersalivation.

## Materials and methods

### Study design and participants

This pilot observational study was conducted on 12 patients diagnosed with PD or atypical parkinsonian syndromes, followed at our specialized outpatient clinic. Inclusion criteria were: male or female subjects ≥ 18 years of age with confirmed idiopathic PD or atypical parkinsonism diagnosis according to the Movement Disorder Society (MDS) criteria, clinically significant sialorrhea defined as a minimum score of 2 on the salivation item of the Unified Parkinson’s Disease Rating Scale (UPDRS) Section II [Activities of Daily Living (ADL)], and no movement disorder-related surgery within 6 months of screening. Exclusion criteria included: severe cognitive impairment (MoCA <10), active oral infections, prior invasive treatments for sialorrhea within 6 months or prior salivary gland surgery, use of drugs interfering with salivary glands’ function (anticholinergics), changes in the therapeutic regimen for PD within 4 weeks before the first visit or during the study, or contraindications to shockwave therapy.

Importantly, all patients received ESWT without any changes to their oral pharmacological regimen throughout the entire observation period, including antiparkinsonian therapies and other concomitant medications that could influence salivary function. None of the participants had received botulinum toxin injections within 6 months prior to enrollment, nor did they receive botulinum toxin at any time during or after the ESWT intervention.

All participants provided written informed consent in accordance with the Declaration of Helsinki. The study was approved by the local university ethics committee.

### Clinical assessment and outcome measures

The study was carried out between April and September 2025 at the Neurology Clinic of Cattinara Teaching Hospital in Trieste.

For each participant, demographic data, disease duration, and clinical scores were collected. Subjects were screened by telephone and asked to rate their baseline condition using item 2 of Section II of UPDRS. Investigators rated salivation from 0 (normal) to 4 (severe drooling requiring constant use of tissues or handkerchiefs).

Patients completed a series of validated questionnaires at baseline and at 1, 4, 8, and 12 weeks after the initial treatment.

Throughout the 12-week follow-up period, patients were asked to keep a detailed daily and hourly diary to monitor the course and fluctuations of sialorrhea symptoms. All assessments and diary reviews were conducted by experienced neurologists.

The primary efficacy endpoint was the improvement of Drooling Frequency and Severity Scale (DSFS) as evaluated by the investigator, which measured both the frequency and severity of drooling. Drooling frequency was rated on a 4-point scale ranging from 1 (never drools) to 4 (constant drooling), while severity was assessed on a 5-point scale from 1 (dry, no drooling) to 5 (profuse drooling with wetting of clothing/objects).

Additional assessments included at baseline and at week 1, 4, 8, and 12 (secondary endpoint):

- *Nocturnal Hypersalivation Rating Scale (NHRS),* rating the severity of nocturnal drooling with scores ranging from 0 (absent) to 4 (hypersalivation wakes the patient up at least 3 times during the night).
- *Sialorrhea Clinical Scale for Parkinson’s Disease (SCS-PD).* A 7-item scale that assesses drooling, physical, and psychosocial domains such as degree of diurnal and nocturnal drooling, difficulties in speaking and eating. The overall score ranges between a minimal score of zero and a maximum of 21, with a higher score indicating higher discomfort.

At baseline and at week 4:

- *Drooling Impact Score (DIS),* evaluating the effect of sialorrhea on daily functions such as speech and social interactions. This is a 10-item questionnaire with overall score ranging from zero to 100, the higher score indicating a more severe impact.

At week 12:

- *Clinical Global Impression of Change (CGI-Change) and Patient Global Impression of Change (PGI-Change)* (1 = very much improved, to 7 = very much worse).

### Shockwave treatment

An electromagnetic coil lithotripter (Duolith Storz Medical AG) under ultrasound guidance was used. Ultrasound imaging was used solely to ensure precise targeting of the salivary glands during ESWT. No ultrasonographic monitoring or morphological evaluation of the glands was performed as these assessments were not included in the study protocol.

**Figure 1:**
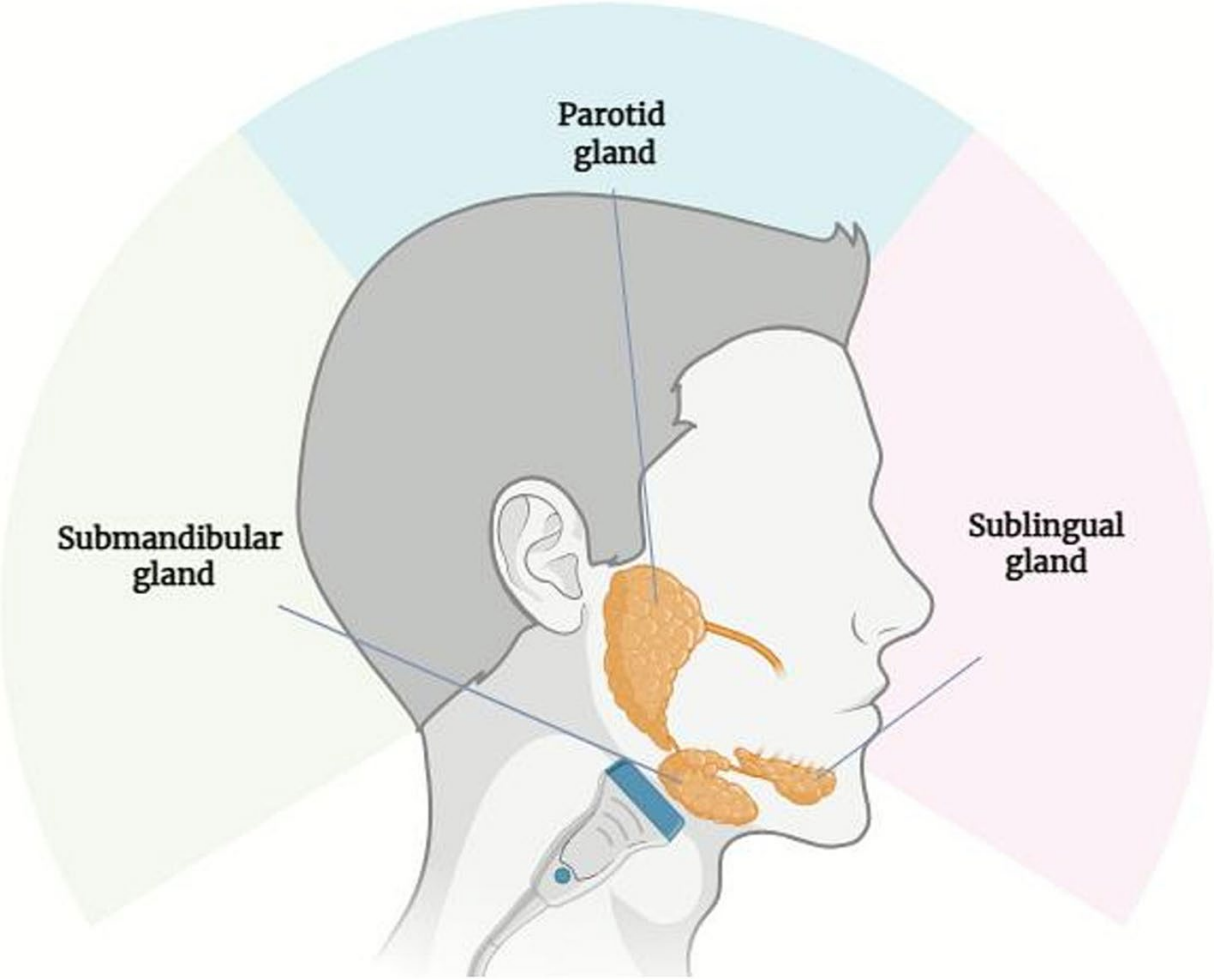
Schematic representation of the major salivary glands. Image created with BioRender.

**Figure 2:**
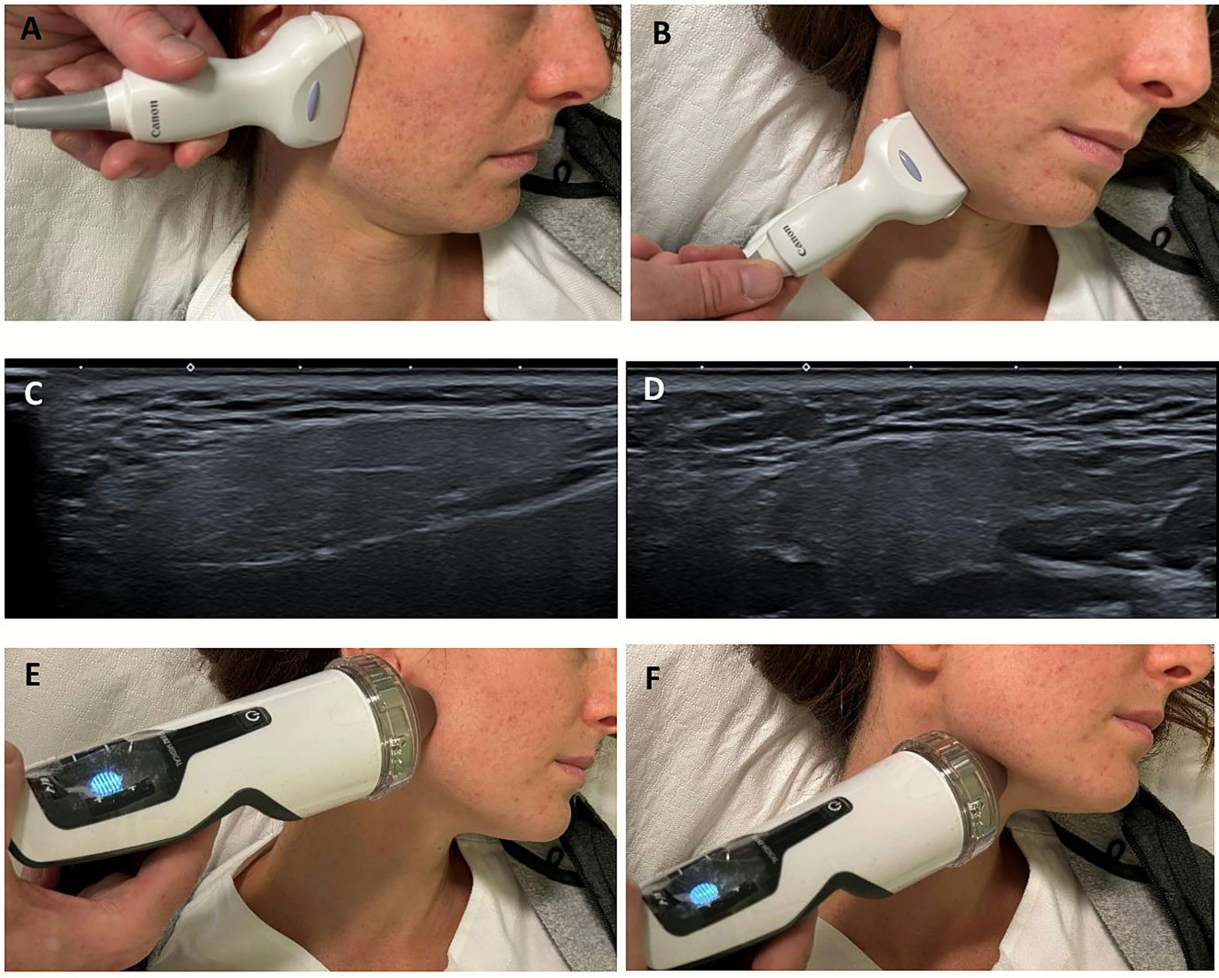
Clinical photographs showing the positioning of the ultrasound probe and the ESWT applicator the over the parotid and submandibular regions **(A,B,E,F)**. Corresponding ultrasound images confirming correct probe placement and real-time visualization of the targeted glandular tissue, parotid **(C)** and submandibular **(D)**, respectively.

The intervention was performed using focal ESWT, applied bilaterally to the major salivary glands (Figures 1, 2). Each patient received 750 impulses per gland, with low level of energy ranging from 0.1 to 0.3 mJ/mm^2^ and a frequency of 5 Hz over the area of the glands. The treatment sites included: right parotid gland: 750 impulses, left parotid gland: 750 impulses, right submandibular gland: 750 impulses, left submandibular gland: 750 impulses. The procedure is painless and does not require anesthesia or analgesics.

The entire protocol was repeated after 1 week (second session), for a total of two treatment sessions per patient.

### Statistical analysis

Shapiro–Wilk test was used to assess the normality of the distribution of continuous data. Continuous variables were presented as median (range) or mean ± standard deviation (SD), according to distribution. Pearson or Spearman correlations were applied to verify the possible correlations between DSFS, NHRS, and SCS-PD scores and clinical variables, as appropriate. Baseline and T2 DIS score were compared with Wilcoxon signed rank test. In order to evaluate the effect of ESWT treatment on clinical scores over time, a general linear model (GLM) for repeated measures was applied including Time (baseline and post-treatment) as a within-subject factor. Statistical analyses were performed using IBM SPSS Statistics 31.0. A *p*-value <0.05 was considered statistically significant.

## Results

Baseline demographic characteristics and clinical features of patients are shown in Table 1.

**Table 1:** Patients’ demographic and clinical characteristics at baseline.

Clinical characteristics	*N* = 12
Sex F, *n*%	2 (16.7%)
Age, y	73.2 ± 9.2
Age at disease onset, y	64.6 ± 9.2
Disease duration, y	8.7 ± 4.3
Most affected side, *n* %	
Right	4 (33.3%)
Left	6 (50%)
Bilateral	2 (16.7%)
Phenotype, *n* %	
Tremor	6 (60%)
PIGD	2 (16.7%)
Mixed	2 (16.7%)
Atypical parkinsonism	2 (16.7%)
Cognitive and motor impairment	
H&Y	2 (2–4)
UPDRS-TOT	72.5 ± 20.7
MoCA corrected	28 (14–30)
Pharmacological treatment	
L-DOPA, *n* %	11 (91.7%)
L-DOPA, mg	420.8 ± 210.5
DA, *n* %	2 (16.7%)
MAO-B, *n* %	7 (58.3%)
COMT, *n* %	2 (16.7%)
Amantadine, *n* %	0 (0%)

According to item 2 of section II of the UPDRS, 10 of the 12 patients reported severe salivation requiring constant use of tissues or handkerchiefs, whereas the remaining two experienced salivation while awake but generally did not require them.

No statistically significant correlations were found between clinical variables and DSFS, NHRS, and SCS-PD scores (data not shown).

**Figure 3:**
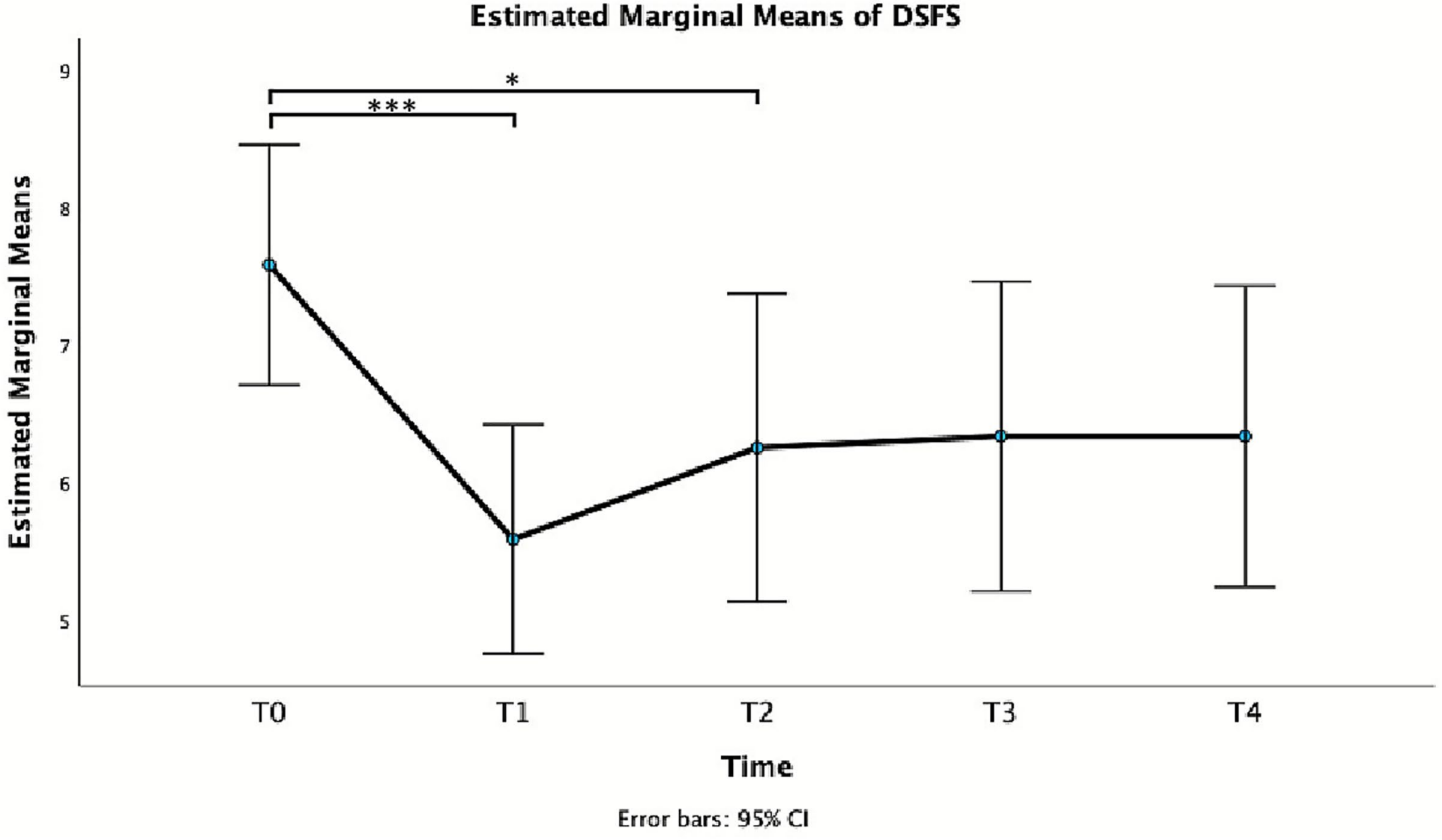
Estimated marginal mean of DSFS.

For DSFS, repeated measures GLM revealed a significant effect of Time, *F*(4, 44) = 6.40, *p* < 0.001, partial *η^2^* = 0.368. In particular, a statistically significant difference was observed between T0 and T1 and T2 (Table 2 and Figure 3), with a reduction of the score at week 1 and 4.

**Table 2:** Comparison between DSFS, NHRS, and SCS-PD at T0 and other timepoints.

Scale	T0 (BL)	T1 (week 1)	T1 *vs* T2		T2 (week 4)	T1 *vs* T3		T3 (week 8)	T1 *vs* T3		T4 (week 12)	T1 *vs* T4	
			Mean diff (CI 95%)	*p*		Mean diff (CI 95%)	*p*		Mean diff (CI 95%)	*p*		Mean diff (CI 95%)	*p*
DSFS	7.58 ± 1.38	5.58 ± 1.31	2.00 (0.86–3.14)	**<0.001**	6.25 ± 1.77	1.33 (0.02–2.65)	**0.046**	6.25 ± 1.78	1.25 (−0.31–2.81)	0.172	6.33 ± 1.72	1.25 (−0.31–2.81)	0.172
NHRS	1.17 ± 0.58	0.33 ± 0.49	0.83 (0.25–1.42)	**0.004**	0.83 ± 0.72	0.33 (−0.32–0.99)	1.000	1.08 ± 1.00	0.08 (−0.72–0.88)	1.000	0.75 ± 0.45	0.417 (−0.26–1.09)	0.538
SCS-PD	12.25 ± 3.79	7.67 ± 4.41	4.58 (1.91–7.25)	**<0.001**	9.25 ± 4.60	3.00 (1.23–4.78)	**0.001**	9.75 ± 4.48	2.5 (0.41–4.59)	**0.015**	9.75 ± 3.96	2.5 (−0.25–1.91)	0.080

Mean ± SD; BL, baseline.

*p*-value < 0.05 statistically significant.

**Figure 4:**
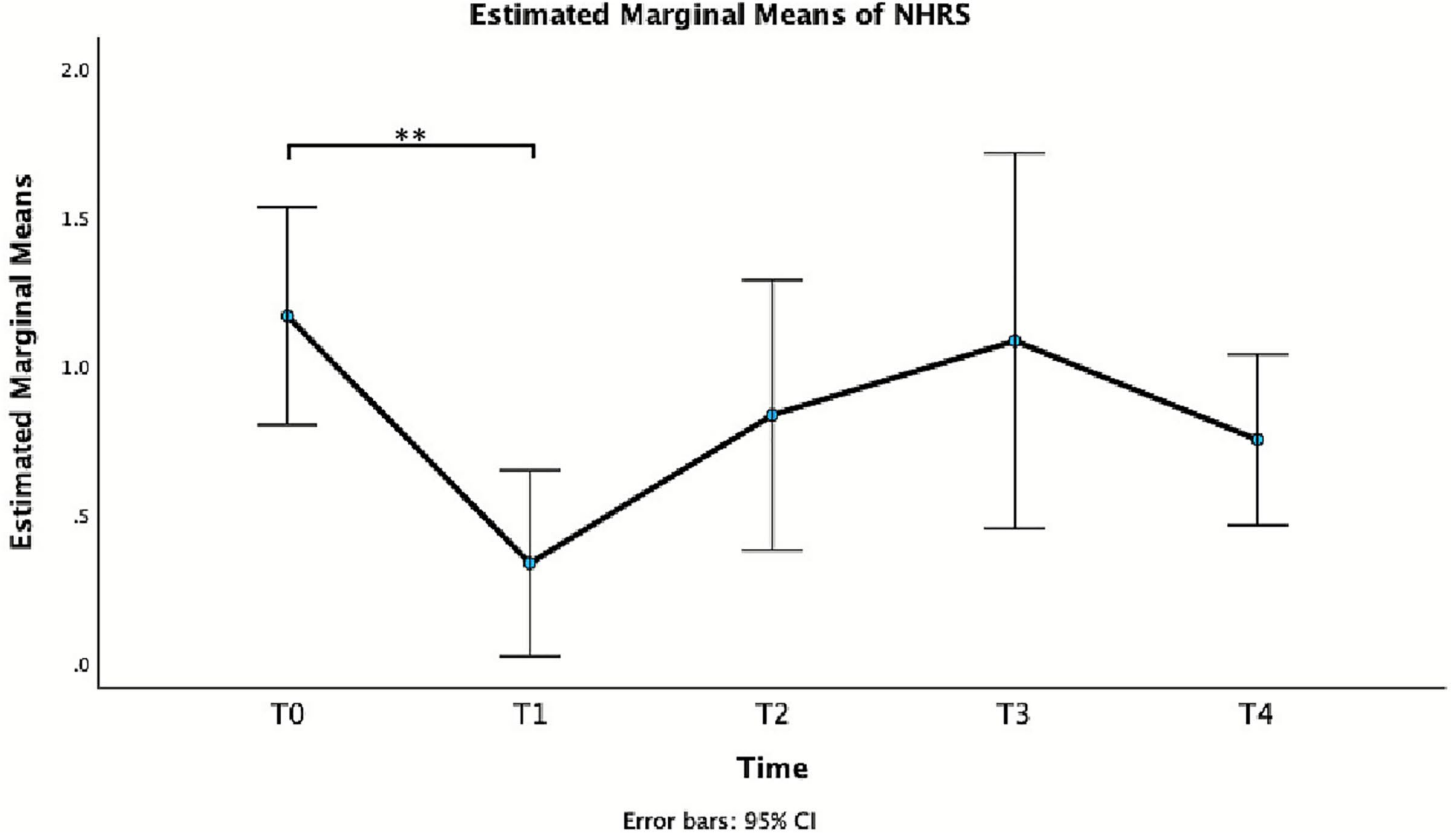
Estimated marginal means of NHRS.

Also for NHRS, repeated measures GLM showed a significant effect of Time, *F*(2.54, 27.92) = 6.16, *p* = 0.004, partial *η^2^* = 0.359. In particular, a statistically significant difference was observed between T0 and T1 (Table 2 and Figure 4), with a reduction of the score at week 1.

**Figure 5:**
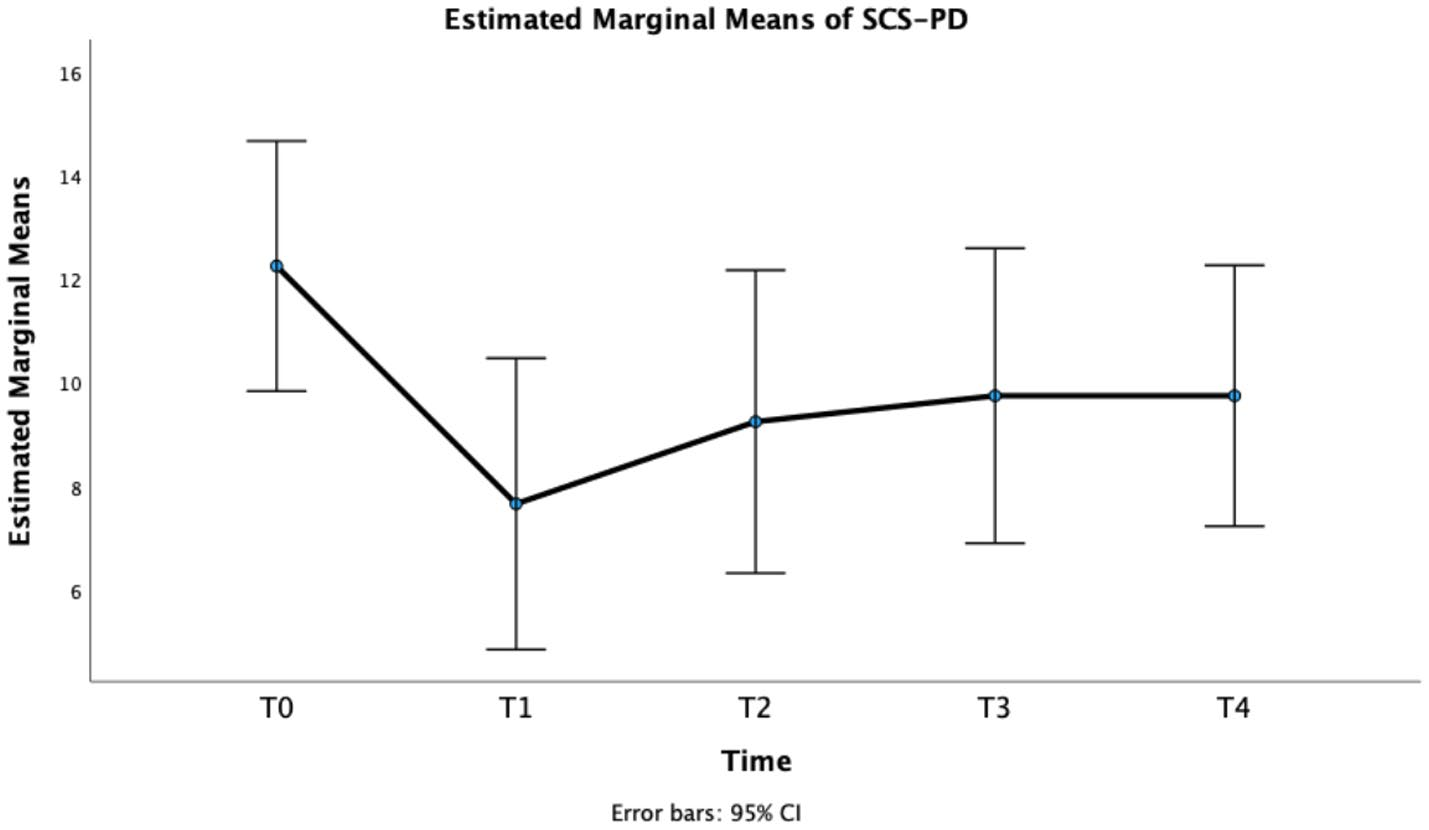
Estimated marginal means of SCS-PD.

Finally, also for *SCS-PD* repeated measures GLM showed a significant effect of Time, *F*(4, 44) = 7.52, *p* < 0.001, partial *η^2^* = 0.406. In particular, a statistically significant difference was observed between T0 and T1, T2, and T3 (Table 2 and Figure 5), with a long-lasting reduction of the score at week 1, 4, and 8.

A decrease in hypersalivation on the PGI-Change during the first month was reported by 75% (9/12) of patients. Of these, 66.7% (6/9) noted only minimal improvement, whereas the remaining three described themselves as “improved” (2/9) or “much improved” (1/9). The remaining three patients did not report any improvement following the treatment. These results were corroborated by the CGI-Change, indicating that 83.3% (10/12) of patients exhibited some degree of improvement in hypersalivation. Of these, 70% (7/10) were rated as minimally improved and 30% (3/10) as improved, while 16.7% (2/12) showed no change.

**Figure 6:**
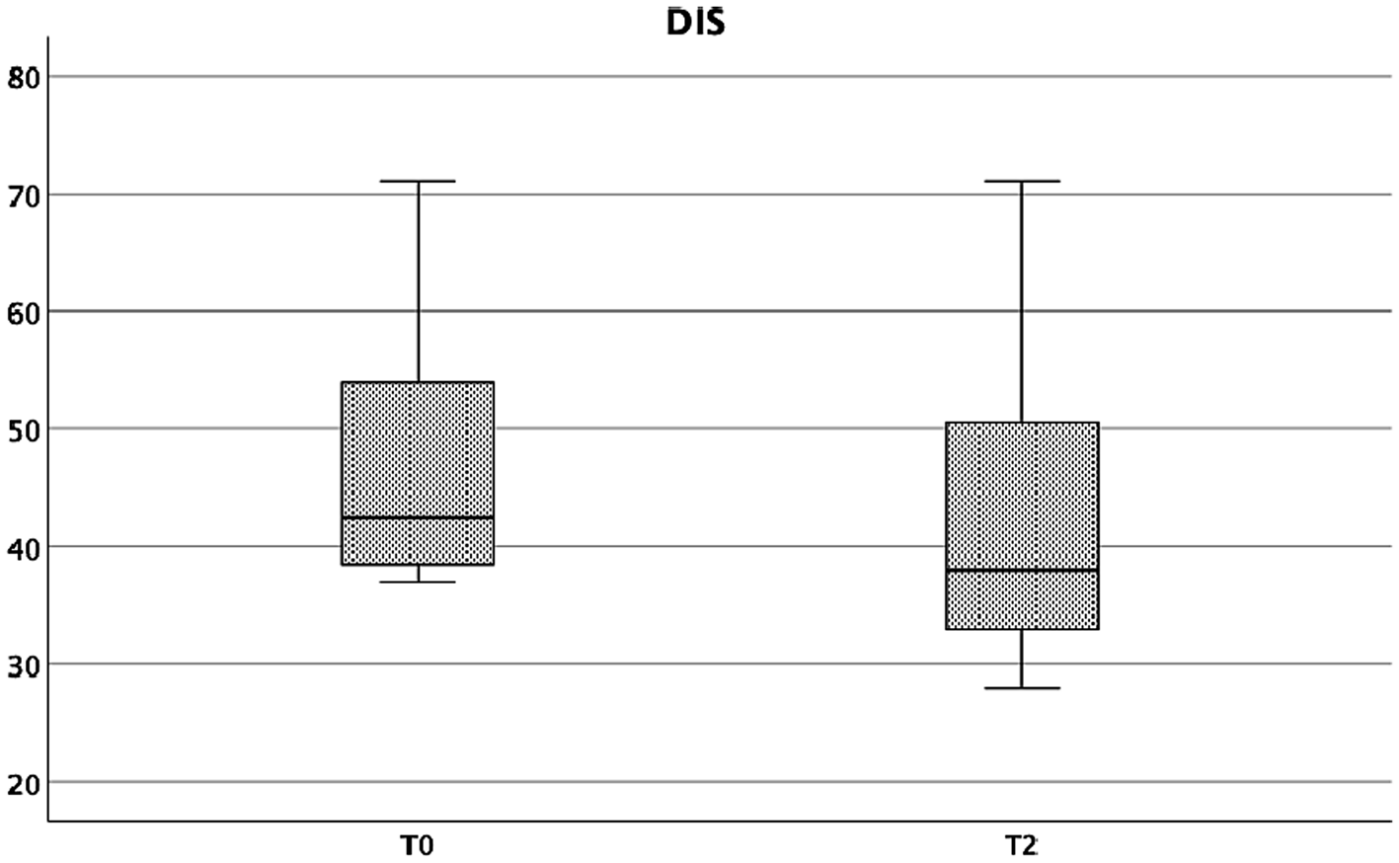
DIS at baseline and at week 4.

Finally, also DIS score showed a statistically significant reduction form baseline to week 4, in particular: T0 = 42.5 (37–71) *vs* T2-week4 = 38 (28–71), Wilcoxon signed rank test, *p* = 0.005 (Figure 6).

## Discussion

The main finding of this study is the significant effect of ESWT in reducing sialorrhea in patients with PD and atypical parkinsonian syndromes, as demonstrated by the clinical rating scales. The effect was evident both in terms of clinical improvement and subjective benefit. The improvement was evident as early as 1 week after treatment and persisted up to 4 weeks when assessed with the DSFS, and even longer, up to 8 weeks, according to the SCS-PD. Importantly, ESWT was well tolerated, and no adverse events or treatment-related discomfort were observed. No patients reported discomfort during stimulation, nor adverse effects such as pain, excessive dryness, or dysphagia. ESWT did not interfere with normal salivation or with saliva production during eating, digestion, or emotional stimuli.

Salivation is a complex autonomically regulated process that represents the initial step in digestion. The major salivary glands (parotid, submandibular, and sublingual) produce between 1 and 1.5 liters of saliva daily. The parotid glands receive parasympathetic innervation via preganglionic neurons in the inferior salivatory nucleus, with project through the glossopharyngeal nerve and lesser petrosal nerve to the otic ganglion (33). In PD, subcortical and brainstem structures involved in autonomic and motor control are affected early, leading to systemic and oropharyngeal bradykinesia (34). This results in impaired swallowing, inefficient clearance of saliva, and accumulation of secretions in the oral cavity. Historically, sialorrhea in PD has been attributed to excessive salivary production secondary to autonomic dysfunction. However, more recent studies demonstrate that saliva output is actually reduced in PD compared to healthy controls (9, 35). Therefore, sialorrhea in PD is most likely due to impaired swallowing, secondary to oropharyngeal bradykinesia, and the inability to clear secretions, leading to excessive pooling of saliva rather than hypersalivation (10). This distinction has therapeutic relevance, as treatments that further suppress salivary secretion, such as BoNT/A injections, may inadvertently worsen swallowing function in vulnerable patients. BoNT/A injections are currently considered the gold standard for sialorrhea management, with robust supporting evidence from clinical trials and case series (14–24). BoNT/A acts by selectively binding to peripheral cholinergic nerve terminals, where it is internalized and inhibits acetylcholine release at the neuroglandular junction, in a dose-dependent but reversible manner (36, 37). Despite proven efficacy and relatively favorable safety profile, BoNT/A injections are invasive, require ultrasound guidance, and can occasionally cause transient dysphagia or xerostomia. These limitations can reduce their applicability in patients with advanced PD or atypical parkinsonism, who often have poor tolerance or cooperation. By contrast, ESWT exerts its effect through local modulation of glandular function without directly impairing swallowing dynamics. Its principal advantage lies in its noninvasive, painless, and repeatable nature—particularly valuable in frail patients at risk of dysphagia or swallowing disturbances due to oropharyngeal bradykinesia.

The relatively long-lasting reduction in sialorrhea observed in this study suggests that ESWT could represent a feasible therapeutic option for this disabling symptom, particularly in patients with advanced PD and atypical parkinsonian syndromes. ESWT has gained increasing application in neurology, particularly in the treatment of spasticity in stroke and CP patients (28, 38). The mechanisms underlying the effects of ESWT on salivation remain speculative. Experimental evidence suggests that low-energy shock waves can modulate cholinergic neurotransmission through a transient reduction in acetylcholine release at the neuromuscular junction. Additionally, ESWT may enhance local nitric oxide (NO) production, improve microcirculation, and exert anti-inflammatory effects at the tissue level (31, 32, 39–41). These effects may collectively downregulate salivary gland activity, producing an anticholinergic-like modulation without systemic exposure and side effects. In this regard, the action of ESWT may parallel the functional mechanisms observed in its use for spasticity reduction (29, 30, 42–44).

This preliminary study represents an initial step in exploring an innovative, palliative, and noninvasive treatment for sialorrhea in advanced PD. Its noninvasive, painless, and repeatable nature enhances patient adherence and tolerability. Although the magnitude of improvement observed in our cohort was modest compared with BoNT/A trials, the absence of adverse effects and the favorable safety profile position ESWT as a promising adjunctive or alternative treatment for sialorrhea, especially in patients at high risk of aspiration or intolerance to invasive procedures. Beyond potential symptomatic relief, reducing drooling may also mitigate secondary complications such as perioral skin maceration, aspiration, and social embarrassment, while improving patients’ social confidence and QoL.

However, several limitations should be acknowledged. The small sample size and open-label, uncontrolled design limit statistical power and generalizibility. Objective assessments of salivary flow, such as sialometry or salivary gland scintigraphy, were not performed. Moreover, potential placebo effects cannot be excluded. These limitations warrant confirmation through larger, multicenter, randomized controlled studies.

## Conclusion and future directions

In conclusion, our findings provide preliminary evidence that low-energy ESWT applied to the major salivary glands is a safe, well-tolerated, and potentially effective noninvasive intervention for sialorrhea in PD and related disorders. ESWT may exert local modulatory effects on glandular activity without compromising swallowing function, making it particularly suitable for fragile or cognitively impaired patients.

In our protocol, ultrasound imaging was employed to guide the treatment application, ensuring accurate targeting of the salivary glands. Although not intended for morphological assessment, ultrasonographic monitoring contributed to the precision and safety of ESWT delivery. Therefore, we suggest that integrating a pre- and post-treatment ultrasonographic evaluation could represent a valuable procedural adjunct for future studies, providing further insight into potential tissue-level effects of ESWT.

Future randomized, double-blind, sham-controlled studies with extended follow-up are needed to confirm efficacy, define optimal treatment parameters, and elucidate the underlying physiological mechanisms. If validated, ESWT could represent an innovative, accessible, and patient-friendly addition to the therapeutic options for the management of sialorrhea in neurodegenerative conditions.

## Data Availability

The raw data supporting the conclusions of this article will be made available by the authors, without undue reservation.
